# Physiological and Moral Management of Infancy

**Published:** 1840-10

**Authors:** 


### ART. XIII.

*A Treatise on the Physiological and Moral Management of Infancy.* By ANDREW COMBE, M.D., &c. &c.—*Edinburgh*, 1840. 8vo, pp. 375.

WE have already recorded our favorable opinion of Dr. Combe's work; and a second and more careful perusal of it amply confirms the impression that the first and necessarily hasty glance we could give it left upon our minds. It is one of the very few books which, addressed principally to parents for their instruction, may be read by medical students and even medical practitioners with great advantage and pleasure. It is true that Dr. Combe does not arrest the attention of his reader by any novel facts. He has drawn freely from various sources for the purpose of rendering his treatise complete, and most admirably and judiciously has he employed the materials he has selected. The style in which the work is written is highly attractive; it is perspicuous and convincing. Every point he urges and every doctrine he inculcates is supported by a reference to well-established physiological facts and to indisputable statistical records. It may be thought, perhaps, by many that in the present day such a work was not required, and it is true, as Dr. Combe admits in his preface, that many excellent treatises on the management of infancy already exist. But most of those hitherto published touch very briefly upon the general management of early childhood, merely as preliminary to a description of its diseases; and their perusal by non-professional readers not unfrequently leads to dangerous tampering with the lives of the young. They are in fact fit guides for medical readers only. Those again, which, as intended for the use of mothers, are free from this objection, lose much of their value and utility from presenting their rules and admonitions as so many abstract and individual opinions, and omitting to connect them with the physiological laws or principles on which they are based, and according to which their effects are produced. Dr. Combe, sensible of these imperfections as detracting from the usefulness as guides for the non-professional reader, and we may add for medical students of many works in other respects of great merit, presents us with the present able treatise on a more comprehensive plan; it avoids on the one hand all descriptions of disease, and on the other founds its precepts on well-ascertained physiological principles. It has been his constant endeavour to allow as little as possible to rest on mere opinion, but to show a foundation for every rule, precept, and injunction in the laws of the human constitution, and consequently in the will of the Creator. "It is then in the habitual application of principle to the inculcation and advancement of knowledge more than in any absolute novelty of detail," that the author hopes the present volume will not be found unworthy of notice. We feel confident that every candid and intelligent reader will determine with us, after a perusal of this little work, that the author's hopes must be fully realized. The chief aim, then, of Dr. Combe in preparing the present treatise is to present the reader with a more comprehensive and systematic view than we usually meet with, of the *principles* by which infant management ought to be directed. It follows, as a necessary consequence, that if these be rightly understood, not only will the details of rules and of general advice be attended to with greater punctuality, but the rules themselves will be fulfilled with more intelligence and with a deeper sense of the responsibility involved in their neglect. It is well known that an immense improvement in the treatment of the young has taken place within the last fifty years, and that as a consequence the rate of mortality in infancy has been greatly reduced. But who will deny that it is equally true that this mortality, although much smaller than formerly, still continues so excessive in amount, as to demonstrate the necessity of still further improvements? "And the more we consider the past the more evident will it appear, that the chief obstacle to our progress arises from trusting too much to random observation, and neglecting the aid and guidance of principle, by which alone observation can be profitably directed and brought to yield its full harvest of results." It is very fairly urged by Dr. Combe that among the numerous works now in the hands of the profession and of mothers, there is not one which attempts systematically to base its rules of conduct on the laws of the infant constitution. And yet it is so plain and natural that every living being should be treated according to its own nature, that we should look with amazement on any one who, on receiving the charge of a new and valuable plant or animal, should proceed to treat it according to his own notions of what is right for plants in general, without first using every means to discover its individual properties and proper mode of management from persons already acquainted with them, and verifying the opinions of such persons by observation of its habits and structure. In practice, this philosophical and even rational mode of proceeding is too often neglected. Instead of invariably consulting nature as the highest authority, we too frequently neglect her dictates altogether, and prefer, or at least carelessly take the mere opinion of the first adviser we meet.

"If we could once bring ourselves to believe that in man, as well as in other animals, adaptation to the laws of the original constitution is the proper standard by which to regulate our treatment of the young, we should derive as much advantage from adhering to it in his instance as we do in that of horses. We acknowledge that the dray and the race horse are different in constitution, and by treating each according to its own nature, we succeed in adapting both to their true purposes. Let us follow the same method with the offspring of man, and similar success will assuredly reward our pains. Such, accordingly, shall be my aim in the following pages, so far as the extent of our knowledge will permit; and where I shall fall short in attaining it, I shall at least have the satisfaction of having done my best to facilitate and render more profitable the exertions of others." (p. 6.)

Thus far for the preliminary observations with which Dr. Combe introduces us to the discussion of many and all of them very important topics connected with the physiological and moral management of infancy. In the abstract and analysis which we now proceed to give of the work, we shall purposely pass over those precepts and instructions which are intended solely for mothers, and confine our notice to those points which we think the medical reader will find neither trite nor unprofitable. And first of the extent of mortality in infancy.

It is a well-known fact that between a third and a half of all the children born die within the first five years of their lives. The presumption naturally arises that some great errors must be committed to entail such a frightful result. And surely this presumption is strengthened when we find no similar fatality among the young of those animals whose structure most nearly resembles that of man, and which are guided in the treatment of their offspring by an unerring instinct which ensures their safety. If, strongly argues Dr. Combe, it were only in wild and barbarous regions that this extraordinary mortality occurred it might seem quite in accordance with the hardships by which even infancy is there surrounded : but the startling circumstance is that it happens in the midst of comfort and civilization, where knowledge and the means of protection are supposed most to abound. As the first efficient step towards preventing or providing a remedy for an evil is to obtain a clear idea of its existence and nature, Dr. Combe refers to the latest statistical records, both foreign and domestic, in proof of the lamentable average mortality of infants which we have just mentioned. Having given unquestionable evidence of the fact, he asks

"Whether this mortality constitutes a necessary part of the arrangements of Divine Providence which man can do nothing to modify; or, on the contrary, proceeds chiefly from secondary causes purposely left, to a considerable extent, under our own control, and which we may partially obviate or render innocuous by making ourselves acquainted with the nature of the infant constitution, and carefully adapting our conduct to the laws or conditions under which its different functions are intended to act?" (p. 11.)

Various considerations are entered into to enable the reader to answer the question for himself. If it can be shown that the preservation or destruction of life in infancy is not of invariable extent, but bears a marked and direct relation to the nature of the treatment and external influences to which the young being is subjected the question will be solved. If the infant mortality be the result of an unalterable dispensation of Providence without respect to good or bad management, we may expect to find it nearly the same in all ages and states of civilization, and bearing no relation whatever to the conduct of others. But if it be chiefly owing to secondary causes, many of which it is in our power to guard against, it will vary in amount, and in direct relation to the favorable or unfavorable circumstances in which the child is placed and the good or bad treatment to which it is subjected. That nothing may be left to uncertainty or conjecture Dr. Combe first contemplates the extent to which in past times infant life *has fallen* a sacrifice to ignorance and bad treatment; and then contrasts it with the comparatively excellent results of a mode of management of a more enlightened though still far from perfect kind.

So directly is infant life influenced by good or bad management that about a century ago the workhouses of London presented the astounding result of twenty-three deaths in every twenty-four infants under the age of one year. For a long time this frightful devastation was thought to be beyond the reach of human remedy. But an improved system was at length adopted, and the proportion of deaths was speedily reduced from 2600 to 450 a year. Other equally striking proofs are advanced of the destruction of infants by bad management, and their comparative preservation by better treatment. From extensive records upon a large scale, and through a long series of years, the most convincing evidence is afforded that it really is in the power of man to prevent and mitigate infant suffering by the enlightened exercise of reason. The grand principle, then, which both parents and medical men ought to have before their eyes, is that human life was not intended to be extinguished at its very dawn, and that when it is so extinguished it is generally from the operation of previously existing causes, some of which might have been discovered and removed, while others might at least have been partially subdued.

*Sources of Disease in Infancy.* All the causes of disease operate by infringing the conditions of health of some organ or organs of the body; and if it were possible to discover the whole of these conditions as affecting *all* the organs, and we could fulfil them scrupulously, we might thereby ward off disease altogether and ensure the continuance of life till the natural term of existence. "The grand aim, consequently, in attempting to improve the treatment of infancy, ought to be the discovery and fulfilment of the conditions on which the healthy action of the principal organs and functions depend." In general the various causes by which health is undermined in infancy will be found to resolve themselves into two distinct classes: those which have reference to the state of the parent before the birth of the child, and those which act directly upon the latter after the commencement of its independent existence.

"On looking abroad upon society, we perceive some families apparently surrounded by every external advantage, yet in which it is found difficult to rear any of the children to maturity. Either from scrofula, consumption, or some other form of bad health, one after another is carried off; and those who survive are characterized by great delicacy of constitution, and require the most assiduous care for their preservation. As a contrast to this, we meet with other families seemingly much less fortunate in their outward circumstances, but in which one child grows up after another as if no such thing as disease existed; or as if the ordinary disorders of infancy were merely mysterious processes for the farther development of the bodily organization. That such remarkable differences exist must have been observed by all who notice what is passing around them; and granting them to exist, the very important question occurs, On what do they depend?" (pp. 56-7.)

The very terms of this statement imply that the unusual susceptibility of disease in the one case and the immunity from it in the other arise from no peculiarity of treatment or external situation, and must therefore depend on some inherent difference of constitution derived from one or both of the parents. So manifest is the influence of hereditary constitution upon the organization and qualities of the offspring that from the earliest ages the attention of mankind has been directed to its observation. The reality of hereditary influence being admitted, the next point of practical importance is to discover what are the conditions in the parents which affect most powerfully the future welfare of the child. They are principally,

"1st. Natural infirmities of constitution derived from their own parents. 2dly. Premature marriages, especially of delicate females, and persons strongly predisposed to hereditary disease. 3dly. Marriages between parties too nearly allied in blood, particularly where either of them is descended from an unhealthy race. 4thly. Great disproportion in age between the parents. 5thly. The state of the parents at the time of conception; and, lastly, The state of health and conduct of the mother during pregnancy." (p. 58.)

Having first adverted to many well-known facts proving the influence of the constitution of the parents on the qualities and health of the progeny, Dr. Combe offers several remarks which are well worthy the attention of medical practitioners. Those who desire bodily and mental soundness in their offspring ought to avoid intermarrying with individuals who are either feeble in constitution or strongly predisposed to any serious disease: as insanity, scrofula, cancer, or consumption; and above all the greatest care should be taken against the union of the same morbid predisposition in both father and mother. If any peculiarity of constitution is confined to one parent, and is not very strong, it may be kept in abeyance by a judicious marriage; but if its influence is aggravated by being common to both parents the children rarely escape. It is not meant that the actual disease which afflicted the parent will certainly reappear in every one of the children, but only that the children of such parents will be much more liable to its invasion than those belonging to a healthier stock, and consequently will require unusual care and good management to protect them against it. One of the chief advantages, indeed, of being aware of the nature and extent of the influence is the power which it gives us of diminishing its operation by a system of treatment calculated to strengthen the weaker points of the constitution. Suppose, for example, a child inherits a scrofulous habit from both its parents, and is brought up under the same circumstances which induced the disease in them, in all probability it will fall a victim to some form or other of scrofulous affection. But exercise timely precaution; transfer the child for a few years to a drier and warmer climate, put it on a proper regimen, and keep it much in the open air, carefully avoiding a damp and unwholesome atmosphere, it may altogether escape the disease, and enjoy better health than its parents.

"A precisely similar result will follow in other cases of family predisposition. The excitable and capricious children of parents who have been insane or are strongly predisposed to become so, will run great risk of lapsing into the same state, if brought up under circumstances tending to increase the irritability of the nervous system, and to call their feelings or passions into strong and irregular activity. Of this description, are excessive intellectual exertion, keen competition at school, over-indulgence, capricious contradiction, and confinement in close warm rooms at home. Whereas, if subjected from the first to a mode of treatment calculated to allay nervous irritability, and give tone to the bodily organization and composure to the mind, the danger in after life may be greatly diminished, and a degree of security enjoyed, which it would otherwise have been impossible to obtain." (p. 64.)

We pass over many excellent comments upon the injurious effects upon the offspring which are derived from the union of parents too nearly allied in blood, from premature marriages, and a great disparity of years in the two parents. We believe very few practitioners are ignorant of the fact, that the state of health and conduct of the mother during pregnancy materially affect the health of the future infant; but we believe also, that from carelessness or compliance with the caprices of females, the knowledge of the abstract fact is practically of little utility because it does not lead to that serious advice and counsel which is likely to be strictly followed. To this very important subject, Dr. Combe devotes an entire chapter. There is no period of life at which it is of so much consequence to observe moderation and simplicity of diet, and to avoid the use of heating food and stimulants, or any powerful and especially sudden mental emotions, as during pregnancy. In regard to regular exercise in the open air, the greatest attention is requisite on the part of the mother. Nothing contributes more especially to a sound state of health during gestation, and to a safe and easy recovery after delivery. With ordinary care, walking may be continued almost to the last hour, and with excellent effect upon all the functions. Every kind of violent exertion should be avoided.

*The Constitution of the Infant at Birth.* So far as the welfare of the child is concerned it may be considered before birth as virtually a portion of the mother's organization. Its life and growth are wholly dependent on her, and it executes no function peculiar to itself. In one sense, indeed, it may be said to carry on growth and nutrition, and to carry on its own circulation; but in reality, all these processes are so closely, though indirectly, dependent upon the mother for their continuance, that they cease with her life, and are affected by every change in her health. The unborn infant being utterly without power to supply any of its own wants, it was the purest benevolence to render it entirely dependent on the parent system, and to deny it any endowment of either feeling or intellect.

"But when the infant is once ushered into the world, what a revolution in its mode of existence takes place! In one instant it is transferred from unconscious repose, solitude, and darkness, to life, and light, and action. From being surrounded by a bland fluid of unvarying warmth, it passes at once to the rude contact of an ever-changing and colder air, and to a harder pressure even from the softest clothing than it ever before sustained. Previously nourished by the mother's blood, it must now seek and digest its own food, and throw out its own waste. The blood, once purified and restored through means of the mother's system, must now be oxygenated by the child's own lungs. The animal heat, once supplied to it from another source, must now be elaborated by the action of its own organs. Formerly defended from injury by the mother's sensations and watchfulness, its own nerves must now receive and communicate the impressions made by external objects; through its smiles or its cries it must now announce to her ear and reveal to her judgment its safety or its danger, and if any of these important changes fail to take place in due time and order, its life may fall a sacrifice." (p. 100.)

For this striking revolution in the mode of life of the new-born infant, its organization must be already prepared at the instant of birth. Dr. Combe gives a brief but very clear and correct sketch of the peculiarities of the infant organization, and the manner in which the necessary changes are brought about. Sensation and muscular motion are the first functions roused into action by the sudden entrance of the new being into the external world. In accordance with this fact, we find the nerves of sensation and motion, and the spinal marrow, from which most of them originate, already firm in structure and largely developed. The anterior and upper portions of the brain, on the other hand, which serve for the operations of the intellectual and moral faculties, then almost in abeyance, are still soft in structure and little developed, while the posterior portion lying behind the ear and connected with the feelings which have for their object the preservation of the individual, and which, therefore, come first into play, is already of considerable size. In strict harmony with this arrangement, the functions of sensibility and muscular motion are *indispensable* to the commencement and continuance of independent existence. "At birth, the lungs and respiratory muscles are, like the well-finished steam engine, quite prepared for action, but like it also they cannot start into activity of themselves." They await the application of the impulse or the moving power, the stimulus of the respiratory nerves. If that stimulus be instantly supplied, breathing will commence; if it be delayed, the lungs will remain inactive and life speedily become extinct. By the shock primarily impressed upon the sensitive nerves of the skin of the new-born infant, by the sudden transition of the infant from a temperature of 98° or 100° in the mother's womb, to one of 60° or 65° in the atmosphere, respiration is first called into action; the same irregular action of the respiratory muscles, the same panting and sighing occurs, which a similar cause, plunging into a cold bath, produces in the adult. Nervous sensibility and muscular motion being thus roused, the next functions called into action for the preservation of life are those performed by the lungs and heart, viz., respiration and circulation. We shall not enter into the detail which the author gives of the mode in which these functions are established, or of their great and important purposes. He renders the subject of the changes which take place in the circulation at birth perfectly easy of comprehension to the general reader and medical student by a clear description and illustrative woodcuts. One peculiarity of the young infant deserves especial notice, namely, the *rapidity* of the circulation. In the first months of life the number of contractions of the heart, and pulsations of the arteries is nearly double that of the adult, and varies from 120 to 130. The breathing also is proportionally frequent, though not deep. It is like the quick short breathing of fever. But as growth proceeds and the chest expands, respiration and the pulsations of the heart diminish in frequency. In infancy, then, we must guard against mistaking a natural for a feverish pulse, and at the same time we must keep in mind that this rapidity of circulation and frequency of respiration, by increasing the nervous excitability, render the system more susceptible of febrile irritation, and hasten the progress of all its acuter diseases.

"From the same peculiarity of the infant constitution, it is obvious that the *purity* of the air in which the child lives must be much more important to its welfare at that age when respiration is imperfect and feeble, than at a later period when the function is more vigorous, and the powers of resistance of the system are greater. In infancy, accordingly, living in a pure dry atmosphere of moderate temperature is the best safeguard of health; and in early life the rapid recovery which often ensues even in very unfavorable circumstances after removal from the confined air of a city to the pure atmosphere of the country, has long been a matter of general observation. For the same reason, the mortality in infancy always bears a direct relation to the impurity of the atmosphere; it is greater in towns than in the country, and in crowded manufacturing districts than in those which are less populous and contaminated." (p. 115.)

Another indispensable condition of life, formerly provided for by the parent, which must forthwith come into play at birth is the supply of animal heat. From various physiological causes, which are explained by Dr. Combe with his characteristic accuracy and clearness, we find that the power of generating heat is feeble in infancy as compared with the adult; while at the same time a regular high temperature of the body is necessary for existence. Hence it follows that whatever withdraws heat faster than it is produced, must be injurious in exact proportion to the extent and rapidity with which the cause acts, and to the natural weakness of the constitution. It should be remembered that so far from infants possessing a power of successfully resisting cold, they, in common with the young of all animals, cannot even sustain their own temperature, and speedily perish unless duly protected externally; and that the degree of animal heat which is indispensable to the continuance of life cannot be kept up till the three great processes of nervous action, respiration, and circulation are fully established.

We cannot follow the author through the whole of the very interesting and instructive outline which he gives of the peculiarities of the infant organization and functions. But we must observe that it admirably conveys to the reader a good general idea of the constitutional tendencies of the young, with which it is so essentially important practitioners should be acquainted, in order that they may direct either their moral or medical management with that confidence and skill which can only result from their having clear and definite notions upon this subject.

Having taken a general view of the peculiarities of the bodily constitution at birth, the author enters upon the more practical part of his enquiry, viz. the consideration of the external conditions and mode of management which experience has shown to be most conducive to the full and regular development of the infant organization, and the preservation of infant health. First, the importance, as a means of health to the young, of a well-situated and well-arranged nursery is considered. The advice the author gives upon this subject is not interesting to mothers only: medical practitioners are not unfrequently consulted upon the selection of the nursery, and for much important information upon the subject we may safely refer them to his guidance. There is so much kindness of feeling as well as of sound wisdom in everything he says upon this subject, that it is with difficulty we refrain from quoting the greater part of the chapter. If we were to judge from the fashions of the day in the clothing of infants and young children, and the indifference with which practitioners in general yield to, or at least do not condemn the many evils that result from its mismanagement, we should infer that whether a young child is or is not properly protected against cold and damp, is an affair of no importance. Dr. Combe shows that the reverse is the fact, and quotes different authorities in support of his opinions. Dr. Eberle, of America, particularly calls attention to the folly and mischief of leaving the neck, shoulders, and arms of children quite bare, while the rest of the body is kept warm; a practice which is generally continued during the first five or six years of life. While adults are so careful to keep these parts so well covered, it is strange that children should be left without protection, not only in winter, but even frequently out of doors in cold and damp weather. Hence, probably, why inflammatory affections of the respiratory organs are so much more common during childhood than at a more advanced age; and that thus, too, the foundation of pulmonary consumption is often laid during the first four years of life. We find from the Reports of Mr. Farr, that young children furnish the majority of cases of pneumonia; of 379 fatal cases of pneumonia in the metropolis, and in some country districts, 228 were children under three years of age. Knowing, however, the disposition to run into extremes, Dr. Combe very properly guards against the opposite danger of loading the child with too many clothes, and covering the shoulders and neck with warm tippets or shawls, even within doors. More mischief may be done by the excessive relaxation thus induced, than by having them exposed. All that is required is, that the ordinary upper dress shall extend sufficiently high to protect the neck and upper part of the chest from variations of temperature. The head is commonly kept too warm in infancy; which, considering the natural tendency to nervous excitement and rapid circulation in early life, is an improper practice. When the head is kept very warm, the nervous excitability is greatly increased: every change makes an impression upon the infant, and any accidental irritation is more likely to be followed by convulsions. Of equal importance to the dress by day is the provision of proper bed-clothing during the night. If an infant is buried under a mass of bed-clothes when asleep, and dressed in the ordinary way when awake, the very transition is apt to be hurtful. Many mothers carefully guard against too much wrapping up by day, who seem to think that at night the health and comfort of the infant depended entirely on the quantity of blankets it could sustain without being smothered.

"In arranging night-coverings, the soft feather-bed is very often estimated as nothing; or, in other words, the same provision of blankets is considered equally indispensable, whether we lie upon a hard mattress, or immersed in down. It is from this confusion that the common mistake above alluded to takes its rise. The mother, looking only to the coverings laid over the child, forgets those on which it lies, although, in reality, the latter may be the warmer of the two. An infant deposited in a downy bed has at least two thirds of its body in contact with the feathers, and may thus be perspiring at every pore, when, from its having only a single covering thrown over it, the mother may imagine it to be enjoying the restorative influence of agreeable slumbers. In hot summer weather much mischief may be done by an oversight of this kind." (p. 185.)

*Food of the Infant at Birth.* Upon this subject we have constantly to contend with the prejudices and "experience" as it is called, of mothers and nurses. The vulgar notion that a child requires to be fed immediately after birth rests on the absurd idea of its having undergone a long fast; whereas, it ought rather to be considered as having just finished a copious meal. During the whole period of its intra-uterine life, it has been supplied with a rich and nutritious blood prepared expressly for its support. Every practitioner of ordinary experience must admit the truth of Dr. Combe's remark, that there is a disposition on the part of mothers and nurses to consider nourishment as, from the first moment of existence, the grand agent which is to avert or cure all possible evils to the child, and thus is the infant stomach constantly oppressed with loads which it is totally incapable of digesting. Many of the "inward fits," "cramps," and "colics," which afflict infancy, owe their origin solely to this cause; and were the laws of the human constitution better known and acted upon, the difficulty of rearing the young would be greatly diminished. When, from the state of the mother, it becomes necessary to administer food to the new-born infant, we should adhere as closely as possible to the intentions of nature, and prefer that kind of nourishment which approaches most nearly to the mother's milk. Were it possible to put the child to the breast of another woman, also just delivered, it would be desirable to do so, but this cannot always be done. The next best food is fresh cow's milk, tepid and diluted with one half of water and slightly sweetened. Gruel, pap, panada, &c. are all improper; the stomach of a new-born child is incapable of digesting any of these articles. Again, too, the quantity of the food is just as important as the quality. A tablespoonful may be given once in three or four hours. If the mother is in health, and the secretion of milk not interrupted, there can be no fair pretence for giving the infant any other food than the mother's milk for several months. Nature unequivocally indicates this truth, by the absence, till a later period, of the organization required for the preparation and digestion of more solid kinds of substance. The next point to determine is, how often may the infant be put to the breast. Here, again, it is the duty of the practitioner, which he too frequently neglects, to guard the parent against excess. If the stomach is too frequently replenished, or too much distended even with the mother's milk, digestion is enfeebled, and gripes and flatulence arise and torment the child. The usual practice with mothers is to offer the breast whenever the child cries or appears uneasy, no matter from what cause, as if hunger were the only sensation which the young being could experience. Crying is considered as an infallible sign of an empty stomach. But

"New as the infant is to the surrounding world, it shrinks instinctively from every strong sensation, whether of heat or of cold, of pressure or of hardness, of hunger or of repletion. Its only way of expressing all disagreeable feelings is by crying. If it is hungry, it cries; if it is overfed, it cries; if it suffers from the prick of a pin, it cries; if it lies too long in the same position, so as to cause undue pressure on any one part, it cries; if it is exposed to cold, or any part of its dress is too tight, or it is held in an awkward position, or is exposed to too bright a light or too loud a sound, it can indicate its discomfort only by its cries; and yet the one remedy used against so many different evils is, not to find out and remove the true cause of offence—but to offer it the breast! No doubt, silence is sometimes obtained by the apoplectic oppression of a stomach thus distended; but no sane being will seriously contend that such quiet is really beneficial, or is such as any mother ought to content herself with procuring." (p. 196.)

On an average, during the first weeks of existence, the child may be put to the breast about every three hours, and as it becomes older the interval may be gradually extended. Even from earliest infancy, regular intervals should, as far as possible, be observed in giving nourishment; and it is surprising how soon the infant accommodates itself to the practice. The quiet repose enjoyed during the interval is beneficial alike to parent and child.

As it sometimes happens that the mother cannot, and sometimes that she will not, suckle her child, a wet nurse becomes necessary, and Dr. Combe enters at some length upon the choice, properties, and regimen of the wet nurse. Artificial nursing, or "bringing the child up by hand," ought never to be preferred as a matter of choice. Strong healthy children may thrive under good management, although denied the breast; but very few delicate children, and still fewer of those born prematurely, survive when brought up by hand. When a child is to be reared by the hand, we have to determine, first, the kind of nourishment; and, secondly, the manner in which that nourishment ought to be given; and upon both these points the young practitioner will do well to consult Dr. Combe. Upon the subject of weaning, too, we find much sensible advice, which we shall briefly notice; for although the general rules as to the mode of weanings, and the period when infants ought to be weaned, are, we doubt not, well understood, we have had many opportunities of knowing that the occasional necessity or at least the advantage of deviating from those rules is very frequently lost sight of. The time of weaning ought to be determined chiefly by two circumstances: the health and state of the mother, and the development and health of the child. When the health of the mother continues perfect, and the supply of milk abundant, weaning ought not to take place till the development of the teeth shows that a change of food is required. This usually happens about the ninth or tenth month; but in delicate children teething is not unfrequently delayed for some months longer, and in such cases, weaning ought not to be permitted so soon. If, however, the supply of milk proves insufficient for the nourishment of the child, and the health of the mother begins to suffer before the expiration of the usual time of suckling, it may become necessary both for the infant's and mother's sake to wean it gradually before any indications of teething arise. In some parts of the continent suckling is continued for eighteen months or two years; but, unless in very feeble and ill-constituted children, this is an unnecessary prolongation of the process, and however well the mother might appear to bear it at the time, it would probably be ultimately injurious to her. In weak scrofulous children, the teeth are frequently late in appearing, and this may be taken as a sure sign that the breast ought still to constitute the chief source of their nourishment. Sir James Clark recommends the children of consumptive parents to be suckled for eighteen months or two years, as the surest means of rendering them healthy and robust; and we believe with Dr. Combe, that the soundness of the principle is borne out by experience, provided, of course, that a sufficient supply of good milk can be obtained for that length of time from a healthy nurse. It is very desirable, if possible, to wean a child in fine weather, when it can be much in the open air; as nothing tends more than such exposure to soothe the nervous irritability so often consequent upon the change. The child should be accustomed gradually to the use of other food, and should be taken from the breast by equally slow degrees. Weaning ought not to be effected while the infant suffers under the irritation of teething or any active disease, as the risk of convulsions or serious intestinal disorder will be thereby greatly increased. If, too, the stomach and bowels of the child become much deranged when weaning is attempted, it is prudent to leave off any other kind of nourishment for a time; again confining it to the mother's milk alone, until by appropriate treatment the digestive organs are placed in a better condition. The ordinary articles of food for the child when it is weaning are well known, but sufficient care is not taken that the transition to a substantial diet is very gradual. Dr. Combe very properly cautions his readers against having immediate recourse to medicine to remedy every little ailment which may appear during the time of suckling or weaning. Unfortunately, a propensity exists, perhaps as frequently with young practitioners as with mothers, to consider disease as an extraneous something thrust into the system, which must be expelled by force before health can be restored, and with which the mode of management has little or nothing to do: whereas, disease is nothing more than an aberration from the regular mode of action of the organization, generally caused by errors of regimen, and very often to be removed by a return to the right course. The consequence of viewing disease as arising from something in the system requiring to be removed is, that on the first symptom of its appearance, medicine is resorted to for its expulsion, and probably calomel administered as a purgative, while the error in diet is left in undisturbed operation. Thus the evil is aggravated instead of being cured, and the constitutions of children are not unfrequently thus ruined by medicine, when they might have been restored to health by proper management without a single dose.

"It is the commonest of all remarks heard in a nursery, that 'the child was uneasy, or griped, or feverish, and I gave it so and so,' without the smallest allusion being made to why it was uneasy or feverish, or whether anything was done to remove the offending cause. In my opinion, a more pernicious habit than that of constantly giving medicine to children does not exist, and I would hold the mother or nurse, who should make frequent use of it without advice, as utterly unfit for the duties imposed upon her." (p. 248.)

We may admit that the language here quoted by Dr. Combe is confined to the nursery, but the mischievous practice that he deprecates has a much wider range.

One chapter is devoted to the subjects of *cleanliness, exercise, and sleep in early infancy.* On all ordinary occasions Dr. Combe prefers pure soft water, without any soap, for the ablutions of young children. He thinks "that soap is generally unnecessary, and frequently hurtful." The manner in which young children are washed is by no means so unimportant as may be thought. The safest and most convenient way of washing the infant is by immersion in a bath, comfortably arranged for the purpose. By this means its wet body is exposed to the air only for a moment, when about to be dried. But if, as is usually the case, the child is placed in a small tub, with the greatest part of the body out of the water, and is washed by laving the water about it with the hand or a sponge, the continued and repeated exposure of its delicate skin to the warm water and cold air alternately is very apt to be followed by chills and other bad consequences. The infant should be quickly and gently rubbed dry with soft napkins, and afterwards with the hand, and then carefully dressed. It is of course the duty of the nurse to wash the child, but the medical adviser surely ought to know the best and safest mode of performing this very necessary duty. On account of the great susceptibility of cold which exists in infancy, and the difficulty with which the system resists the influence of any sudden change, the temperature of the water ought, at first, to be nearly the same as that of the body; namely, about 96° or 98° Fahrenheit, and always to be regulated by a thermometer as the only sure test. If the nurse judge by the hand alone, she will often commit an error of several degrees, according to the varying state of her own health and sensations. The younger the infant, the more rigidly should this standard be adhered to; as it is not till after growth and strength have made some progress, that it becomes safe to reduce the temperature by a few degrees. In addition to the regular morning ablution, the tepid bath should be repeated every evening for a few minutes.

"Properly managed, and not too warm, it has the double advantage of soothing the nervous system, which is always irritable in infancy, and of sustaining an equable circulation of the blood towards the surface, and thus warding off internal disease. It ought not, however, to be either too long continued or used in a cold room. With these precautions, the most unequivocal advantage often results from its use, especially in scrofulous and delicate children. For restless and irritable children also, the evening bath is often of immense advantage, from the quiet and refreshing sleep which it rarely fails to induce. As a sedative too, it is of great value in subduing nervous excitement. But when used too warm, or continued too long, the bath is apt to excite undue perspiration, and to increase the liability to cold." (p. 255.)

If from mismanagement, or any other cause, the child is frightened by immersion in warm water, or if the bath decidedly disagrees with it, simple washing or sponging with tepid water should be substituted. Some physicians prefer the cold to the tepid bath, even for infants, but reason and experience concur in condemning it; and unless the infant is of a very strong constitution, it rarely bears the cold bath without injury. In a few months the temperature of the water may be gradually reduced for the *morning* ablution, providing the child is healthy and the weather warm.

*Exercise in infancy* is as essential to health as at any period of life. To regulate it properly, we have only to keep in view the state of the infant organization and the laws under which the principal functions operate. At birth there is no desire for voluntary motion, and no will to direct it. In ordinary cases it is not till the sixth or seventh month that the bones, ligaments, and muscles become solid and powerful enough to support the burden of the head, or to fit the child for sustaining itself in a sitting or erect position. For some weeks after birth, then, exercise should be of a purely passive kind, and we ought not to excite the child to premature exertion, or place it in a sitting or erect position. From neglecting this precaution the soft and yielding spine often bends under the weight of the upper part of the body, and permanent deformity is produced, and as a necessary consequence undue pressure upon the lungs, heart, and digestive organs, and disorder of their respective functions. Hence in the beginning of life, exercise ought to consist simply in being carried about the nursery, or into the open air, in a horizontal or slightly reclining position on the nurse's arms, or in a carriage, and in gentle friction with the hand over the whole surface of the body and limbs—an operation which is not less agreeable to the infant than beneficial in promoting a free and equal circulation. If the child is born in summer or late in spring, its exercise should be confined to the limits of the nursery and adjoining rooms for about a fortnight; it may then be cautiously carried out in the open air for half an hour at a time. "If it is born in winter or late in autumn, it ought not to be taken out till after the lapse of three or four weeks, and then only in fine mild weather, and for a short time." In proportion as the organization of the child becomes developed, and its capabilities increase, it begins to show active desires, and wishes of its own which require a corresponding modification in its treatment. We can only notice one of the many useful hints Dr. Combe offers upon the subject of infantile sleep. Many nurses and mothers imagine, and practitioners if they know often fail to correct the mischievous error, that it is of no consequence how noisy the nursery is while a child is sleeping, provided the child is not roused up broad awake. But it is of consequence. If the room is not quiet the child's sleep is neither sound nor refreshing, and thus is frequently (we speak from experience of the fact) laid the foundation of that extreme nervous susceptibility which renders attacks of convulsions so likely to occur from various occasional causes, that would with more prudent management be escaped from with impunity.

"There are few things which distress an anxious mother or annoy an impatient nurse more than sleeplessness in her infant charge, and there is nothing which both are so desirous to remove by the readiest means which present themselves. A healthy child properly treated, and not unduly excited, will always be ready for sleep at the usual time; and when it appears excited or restless, we may infer with certainty that some active cause has made it so, and should try to find out and remove it. If no adequate external cause can be discovered, we may infer with equal certainty that its health has in some way suffered, and that it is sleepless from being ill. In this case, the proper course is to seek professional advice, and to employ the means best adapted for the restoration of health, after which sleep will return as before. From not attending to the true origin of the restlessness, however, and regarding it merely as a state troublesome to all parties, many mothers and nurses are in the habit of resorting immediately to laudanum, sedative drops, poppy syrup, spirits, and other means of forcing sleep, without regard to their effects on the disease and on the system; and are quite satisfied if they succeed in inducing the appearance of slumber, no matter whether the reality be sleep, stupor, or apoplectic oppression. The mischief done in this way is inconceivably great, and astonishment would be excited if it were generally known what quantities of quack 'cordials,' 'anodynes,' and even spirits, are recklessly given with the view of producing quiet and sleep." (pp. 280-1.)

Passing over many good comments upon the subjects of teething, weaning, and errors in diet, we come to one point which we cannot refrain from noticing, because from what we almost daily witness in the families of medical practitioners, they need the advice given by the author, as much as mothers to whom he more particularly directs it. Nothing is more common than for the greatest anxiety to be felt as to the regularity with which young children take their meals, at stated hours in the nursery, *but*, the same children are often brought to table at the end of their parents' dinner, and wine, fruit, and confections given them, when nothing but mischief can follow such indulgence. If the child is denied the gratification of partaking of what he sees freely granted to all around him he becomes fretful and discontented.

"In their conduct towards children, parents ought never to forget the fundamental principle, that every faculty is roused into action by the presence of its own objects, without any intermediate operation of either reason or judgment; and that both appetite and feeling may be thus cherished and strengthened simply by reiterated exercise. The prayer, 'Lead us not into temptation,' recognizes this truth in a very pointed manner, and attaches to it not more importance than it deserves. Many an individual remains pure and virtuous in the absence of the object of temptation, who would find his powers of resistance taxed to the uttermost by its presence. What, then, are we to think of the wisdom of those who, convinced in their own minds of the impropriety of unduly cultivating the appetites of their children, nevertheless unfairly subject them to temptation by bringing them into contact, after their own whims, with the jellies, puddings, and fruits, which were never intended for them? If the children are brought to table and yet denied a share in these good things, they naturally feel themselves wronged and harshly treated in having desires excited which are not to be gratified. If, on the other hand, after having received their regular meals, they are allowed to partake in another, of which the system does not stand in need, the first result will be the improper pampering of a false appetite, and the second most probably a fit of indigestion. As a general rule, therefore, children ought never to see either food or delicacies, except what are intended for their own use, and at their regular meals; and the practice of giving biscuits, sugar-plums, cake, &c., between meals, ought to be positively forbidden. Even very young children are sufficiently clear-sighted to perceive, or at least to feel, inconsistencies in the conduct of their parents, and cannot understand why, if it is right to give them sweetmeats or wine one day in addition to their ordinary fare, it should be wrong to do so another, or every day. If, again, it is wrong to do so at any time, the confidence of the child in the truthfulness and consistency of the parent is naturally shaken, when he finds the latter in any instance deliberately doing that which he as deliberately condemned." (pp. 309-11.)

The last chapter of the work treats briefly on the moral management of infancy, and is, as we stated in our last number, full of admirable and not a few novel observations.

We have dedicated much more space to Dr. Combe's work than treatises which are chiefly addressed to the public generally deserve or require. But we have found in it so much useful information for the medical reader upon subjects which practitioners very much neglect, that we could not dismiss it more briefly. We know from experience how difficult it is to command the attention of medical students to the subject of the general management of infants and children, or a detailed account of the business of the nursery. It cannot however be denied that it is as incumbent upon them to be as well acquainted with these topics as with infantile diseases, in order that they may be prepared to correct the numerous and glaring errors which are hourly committed by inexperienced mothers and "experienced" nurses. Medical practitioners, in general, confine themselves too much to the mere art of prescribing medicines for children; if they were equally attentive in prescribing for those who have the charge of them a correct and steadily-pursued system of management, they would ward off altogether many of the most serious and common ailments of infants and children, and thus achieve what is our duty, and ought to be our wish, namely, the prevention as much as the cure of disease. They who are desirous of obtaining information upon these very important subjects can consult no better, no safer, nor more agreeable guide than Dr. Combe; nor can they perform a kinder act to children than that of strongly recommending his work to parents.

---

